# Pregnancy associated plasma protein-A2 (PAPP-A2) and stanniocalcin-2 (STC2) but not PAPP-A are associated with circulating total IGF-1 in a human adult population

**DOI:** 10.1038/s41598-024-52074-8

**Published:** 2024-01-20

**Authors:** Katharina Nimptsch, Elif Ece Aydin, Rafael Francisco Rios Chavarria, Jürgen Janke, Matthew N. Poy, Claus Oxvig, Astrid Steinbrecher, Tobias Pischon

**Affiliations:** 1 Molecular Epidemiology Research Group, Max Delbrück Center for Molecular Medicine in the Helmholtz Association (MDC), Robert-Rössle-Straße 10, 13125 Berlin, Germany; 2 Charité - Universitätsmedizin Berlin, Corporate Member of Freie Universität Berlin and Humboldt-Universität zu Berlin, Berlin, Germany; 3 Biobank Technology Platform, Max-Delbrueck-Center for Molecular Medicine in the Helmholtz Association (MDC), Berlin, Germany; 4 Core Facility Biobank, Berlin Institute of Health at Charité - Universitätsmedizin Berlin, Berlin, Germany; 5 John Hopkins University, All Children’s Hospital, St. Petersburg, FL USA; 6 Department of Molecular Biology and Genetics, Aarhus University, Aarhus, Denmark

**Keywords:** Biomarkers, Endocrinology

## Abstract

The pappalysins pregnancy associated plasma protein-A (PAPP-A) and -A2 (PAPP-A2) act as proteinases of insulin-like growth factor-1 (IGF-1) binding proteins, while stanniocalcin-2 (STC2) was identified as a pappalysin inhibitor. While there is some evidence from studies in children and adolescents, it is unclear whether these molecules are related to concentrations of IGF-1 and its binding proteins in adults. We investigated cross-sectionally the association of circulating PAPP-A, PAPP-A2 and STC2 with IGF-1 and its binding proteins (IGFBPs) in 394 adult pretest participants (20–69 years) of the German National Cohort Berlin North study center. Plasma PAPP-A, PAPP-A2, total and free IGF-1, IGFBP-1, IGFBP-2, IGFBP-3, IGFBP-5 and STC2 were measured by ELISAs. The associations of PAPP-A, PAPP-A2 and STC2 with IGF-1 or IGFBPs were investigated using multivariable linear regression analyses adjusting for age, sex, body mass index and pretest phase. We observed significant inverse associations of PAPP-A2 (difference in concentrations per 0.5 ng/mL higher PAPP-A2 levels) with total IGF-1 (− 4.3 ng/mL; 95% CI − 7.0; − 1.6), the IGF-1:IGFBP-3 molar ratio (− 0.34%; 95%-CI − 0.59; − 0.09), but not free IGF-1 and a positive association with IGFBP-2 (11.9 ng/mL; 95% CI 5.0; 18.8). PAPP-A was not related to total or free IGF-1, but positively associated with IGFBP-5. STC2 was inversely related to total IGF-1, IGFBP-2 and IGFBP-3 and positively to IGFBP-1. This first investigation of these associations in a general adult population supports the hypothesis that PAPP-A2 as well as STC2 play a role for IGF-1 and its binding proteins, especially for total IGF-1. The role of PAPP-A2 and STC2 for health and disease in adults warrants further investigation.

## Introduction

The growth hormone (GH)-insulin-like growth factor (IGF) axis has been recognized since decades as it plays a central role in human growth and metabolism^1,2^. The two peptide hormones IGF-1 and IGF-2 are central members of this system. While IGF-2 plays a major role in prenatal development, the growth hormone/IGF-1 axis plays an important role in postnatal growth^3–6^. The primary physiological function of IGF-1 in adulthood is regulating cellular proliferation, differentiation, and apoptosis^4,7^. However, higher circulating IGF-1 concentrations in adults have been associated with higher cancer risk^4,8^, recently supported by Mendelian Randomization studies, particularly for breast, colorectal and prostate cancer^9–12^. IGF-1 in adults has also been associated with metabolic diseases, such as atherosclerosis and type 2 diabetes^13,14^. Further, IGF-1 plays also an important physiological metabolic role in adults, particularly for glucose homeostasis^15^. In humans, the vast majority (approximately 98%) of circulating IGF-1 is bound to one of six binding proteins (IGFBPs)^4^ in either tertiary (IGFBP-3 or IGFBP-5) or binary complexes^16^, with over 90% of IGF-1 being bound to IGFBP-3^6,17^. The IGFBPs may modulate IGF-1 action, by decreasing the free and bioavailable IGF-1 fraction on the one hand and by prolonging the half-life of this protein on the other^18^. It has been described that the half-life of IGF-1 bound in ternary complexes with IGFBPs and an acid labile subunit (ALS) can be as long as 12–16 h, compared to a half-life of less than 10 min of free unbound IGF-1^16,19^.

**Figure 1:**
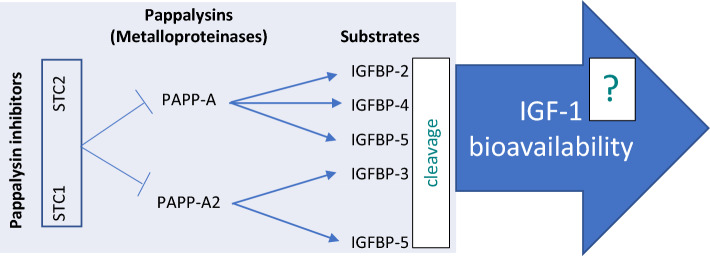
Schematic overview of the stanniocalcin-pappalysin-IGF-1 axis.

Pregnancy associated plasma protein-A (PAPP-A) and PAPP-A2 are members of the pappalysin family of metalloproteinases that cleave insulin like growth factor binding proteins, which may potentially increase IGF-1 bioavailability (Fig. 1). PAPP-A2 cleaves IGFBP-3 and -5^20,21^, thereby releasing IGF-1 from its ternary complex with IGFBP-3 or -5 and ALS^22–24^. PAPP-A has the ability to cleave IGFBP-4, IGFBP-2 and IGFBP-5 and exerts its proteolytic properties primarily at the cellular level, i.e. close to the IGF-1 receptor^25^. In contrast to PAPP-A, PAPP-A2 is present in a soluble form^16^. Initial studies suggested that PAPP-A and PAPP-A2 primarily play a role in pregnancy, since they are highly expressed in the placenta^1,23^, but they are also expressed in other human tissues and cell types^2^. Experiments have shown that mice deficient in PAPP-A or PAPP-A2 show decreased birth weight and postnatal growth retardation^26–28^. In humans, genetic data have pointed at PAPP-A and PAPP-A2 as determinants of human height^29^, and dysfunction of PAPP-A2 was shown to cause short stature^30^. We previously found PAPP-A2 concentrations measurable in a general adult population of men and non-pregnant women^31^. In our analysis, PAPP-A2 concentration was slightly higher in women than in men, and positively correlated with age. It was inversely associated with body mass index and weight, and positively associated with γ-glutamyl transferase, aspartate transaminase and lactate dehydrogenase.

PAPP-A and PAPP-A2 may be important regulators of IGF-1, given their proteolytic cleavage activities on the IGFBPs observed in experimental models. More recently, stanniocalcin (STC) has been described as another player in the pappalysin-IGFBP-IGF-1 axis. The widely expressed stanniocalcins STC1^32^ and STC2^33^ act as potent inhibitors of the proteolytic activities of PAPP-A and PAPP-A2^2,34^ and especially STC2 has been shown to affect IGF-1 actions^35^.

Two studies have described cross-sectional associations between pappalysins, IGFBPs, stanniocalcins and IGF-1 in children, adolescents and young adults^25,36^. In a cross-sectional study of US children and adolescents between 3 and 18 years, PAPP-A2 levels correlated inversely with total IGF-1 and positively with the percentage of free to total IGF-1^36^. Similarly, in a more recent study in newborns and individuals aged 1–30 years from Spain, PAPP-A2 concentrations correlated inversely with total IGF-1 and positively with the free/total IGF-1 ratio^25^. In the same study, STC2 correlated weakly positively with total IGF-1, free IGF-1 and IGFBP-5 and weakly inversely with IGFBP-2. The association of circulating PAPP-A, PAPP-A2 and STC2 with IGF-1 and its binding proteins has not previously been described in a general adult population. Knowledge on these relationships is required to be able to understand the dynamics of this network of molecules and ultimately understand how it contributes to the regulation of IGF-1 signaling, which has important health implications in adults.

The aim of our study was, therefore, to examine the association of circulating PAPP-A, PAPP-A2 and STC2 with free and total IGF-1 and various IGFBPs, in a general (non-pregnant) adult human population.

## Methods

### Study population

The study population is based on two cross-sectional pretest studies conducted from 2011–2012 at the Max Delbrück Center for Molecular Medicine (MDC) to support the implementation of the German National Cohort Study (GNC)^37^, which has been reported previously^31^. Participants in these pretests were sampled mostly randomly via residents’ registration offices (in the first pretest, 34.6% of participants were recruited as a convenience sample, e.g. via e-mail lists) from the general population aged 20–70 years living in the vicinity of the study center. Inclusion criteria were the principal residence in the recruitment area, German language skills and the ability to provide informed consent. All potential participants were invited to take part in a 3-h examination at the study center, consisting of an interview, physical examinations, and a blood draw. Participants were not specifically asked to be fasting for the blood draw. The study protocols were approved by the ethics committee of the Charité University Medicine Berlin and by the data protection officer. All participants gave written informed consent. All examinations were carried out in accordance with the relevant guidelines and regulations.

### Anthropometric data and blood sampling

Weight, height, waist, as well as hip circumference were measured by trained staff following the WHO protocol^38^. BMI was calculated as weight divided by height squared (kg/m^2^). Whole blood, serum and EDTA plasma were collected during the participants’ visit to the study center in 2011/2012. For EDTA, samples were collected in EDTA tubes (Sarstedt Monovette^®^), turned twice, set on a universal rocking mixer for a maximum of 5 min. Serum was collected in serum tubes (Sarstedt Monovette^®^), turned twice and stored upright for 30–45 min. Subsequently, both serum and plasma tubes were centrifuged at 2000 g for 15 min, and the serum or plasma supernatant was aliquoted and immediately frozen on dry ice and stored at − 80 °C^31^.

### Laboratory analyses

PAPP-A2 was measured in plasma samples in one charge using ELISA (AL-109, Ansh Labs, TX, USA)^31^ in the laboratory of Claus Oxvig, and interassay coefficient of variation (CV) was < 15% according to the provider. PAPP-A and STC-2 concentrations were measured by BioVendor Laboratories (Brno—Řečkovice, Czech Republic) in serum with the BioVendor PAPP-A Ultra-sensitive ELISA kit in the case of PAPP-A and the BioVendor Stanniocalcin-2 ELISA kit for STC-2 with inter-assay CVs of 5.5% for PAPP-A and 5.7% for STC-2. Total IGF-1, and IGFBPs were measured in plasma samples in duplicate by BioVendor Laboratories (Brno, Czech Republic) in one charge using ELISAs from BioVendor for IGF-1 (calibrators traceable to World Health Organization IGF-I preparation NIBSC code 02/254, version 6.0), IGFBP-1, IGFBP-2 and IGFBP-3 and an ELISA for IGFBP-5 from Thermo Fisher Scientific™ (Frederick, Maryland, USA). The inter-assay CVs were on average 1.9% for IGF-1, 1.6% for IGFBP-1, 1.6% for IGFBP-2, 1.1% for IGFBP-3, and 2.0% for IGFBP-5. Serum free IGF-1 concentrations were measured in the laboratory of the Molecular Epidemiology Research Group (MDC) using an ELISA from Ansh Laboratories (Webster, TX, USA). Values below the limit of detection (LOD) were observed in certain proportions of the study population using the ELISAs for free IGF-1 (14% < LOD), IGFBP-1 (8% < LOD), IGFBP-5 (68% < LOD) and PAPP-A (27% < LOD).

### Statistical analysis

We excluded 5 participants with missing data in socio-demographic factors, 1 participant with PAPP-A2 concentration 10-times the standard deviation over the mean as well as 5 participants with missing PAPP-A measurement and 3 participants with missing STC2. The final sample included 394 participants. However, for free IGF-1 as dependent variable, the analyses were restricted to n = 339 participants with concentrations above the detection limit, for IGFBP-1 they were for the same reason restricted to n = 360 participants; and for IGFBP-5, they were restricted to n = 125 samples. For PAPP-A, n = 288 participants had samples above the detection limit. In order to use the information from the PAPP-A measurement in a transparent way, for the analysis of PAPP-A as independent variable we applied three approaches: First, a categorical approach where the participants with measured PAPP-A below the detection limit were placed in the lowest category; second, in continuous analyses only PAPP-A values above the detection limit were used; third, in continuous analyses measurements below the detection limit were assigned plausible minimal values (half the lower limit of detection).

Four sampling time point groups from Pretest-1 and Pretest-2 were observed in the data and determined to be used for the later adjustments: One sampling time point group of Pretest 1 (samples and data were collected between August-December, 2011) and 3 sampling time point groups of Pretest 2 (samples and data for the first group were collected between June and July, for the second group between August and September, for the third group between October and December, 2012).

We calculated the percentage of free to total IGF-1 as a measure of the regulation of IGF-1 and its interaction with its binding proteins^36^, which can be seen as a measure of potential ability of IGF-1 to bind its receptor, i.e. a potential proxy for IGF-1 bioavailability. In addition, the IGF-1:IGFBP-3 molar ratio (as percentage) was calculated since it has also been suggested as measure representing IGF-1 bioavailability^15,39–44^. IGF-1 and IGFBP-3 concentrations were converted from ng/mL into nmol/L by multiplying IGF-1 with 0.130 and IGFBP-3 with 0.036 as described in a comparable study^40^ where similar measurement methods were used, and the molar ratio was then calculated by dividing IGF-1 by IGFBP-3.

Participants’ characteristics and median concentrations of measured biomarkers were examined descriptively in the total study population as well as by sex. Based on the histograms and the QQ-plots, the variables, which were (graphically) not normally distributed, were log-transformed to obtain a comparable distribution, i.e. IGFBP-1, IGFBP-5, free IGF-1, free-to-total IGF-1 ratio as percentage and PAPP-A were log-transformed.

Correlation analyses and multivariable regression analyses were performed to study the association of PAPP-A, PAPP-A2 and STC2 concentrations with circulating members of the IGF-1 and its binding proteins as well as age. Partial Pearson correlations coefficients were calculated, controlling for age (years), sex, BMI (kg/m^2^) and pretest (4 sampling time points). We divided the study population according to age- and sex-specific quintiles of PAPP-A2 and STC2 as well as in five categories of PAPP-A (below detection limit as first category and age- and sex-specific quartiles for participants with values above the detection limit). Linear regression models were used to evaluate the association between PAPP-A, PAPP-A2 and STC2 (continuously and across categories) and free and total IGF-1 as well as all measured IGFBPs separately, calculating β coefficients and 95% confidence intervals (CI), adjusted for age (years), sex, BMI (kg/m^2^) and pretest (4 sampling time points). Because it has been shown that IGFBP-1 concentrations are affected by fasting status^45^, models investigating IGFBP-1 were additionally adjusted by fasting status (≥ 6 h, yes vs. no). Based on these regression models, we calculated multivariable adjusted mean values (least squares means) of IGF-1 and IGFBPs by PAPP-A categories, PAPP-A2 quintiles and STC2 quintiles. Furthermore, tests for linear trends across categories were performed by including the category medians as one variable in the models for each dependent variable and presenting the *p*-value for this trend variable. In order to investigate which members of the STC2-pappalysin-IGF-1 system are most relevant for observed associations, we mutually adjusted for different members of this system in the multivariable models. As sensitivity analysis, we repeated the multivariable analyses with exclusion of participants who reported a diagnosis of either osteoporosis, diabetes, autoimmune disease or anti-inflammatory diseases (total n = 43).

Two-sided *p* values of less than 0.05 were considered statistically significant. The multiple linear models were assessed regarding their appropriateness (whether the assumptions of the multivariable linear regression model were fulfilled) using regression diagnostic tools. Statistical analyses were performed with SAS^®^ Enterprise Guide^®^ 7.15 (SAS Institute Inc., Cary, North Carolina, USA).

## Results

Characteristics of the study population are displayed in Table 1. Of the 394 participants, 60% were female, and 40% male, and age ranged from 20 to 69 years (mean age overall 48.9 years, SD 13.8 years, Table 1).

**Table 1:** Characteristics of the study population (n = 394).

Variable	All (n = 394)	Women (n = 235, 60%)	Men (n = 159, 40%)
Age, years, mean (SD)	48.9 (13.8)	48.5 (13.7)	49.5 (13.9)
Height, cm, mean (SD)	171 (9.4)	166 (7.2)	178 (7.2)
BMI, kg/m^2^, mean (SD)	26.0 (5.0)	25.2 (4.8)	27.2 (4.9)
Fasting at blood collection (≥ 6 h), n %)	48 (12.2)	23 (9.8)	25 (15.7)
Self-reported osteoporosis diagnosis, n (%)	13 (3.3)	11 (4.7)	2 (1.3)
Self-reported diabetes diagnosis, n (%)	19 (4.8)	10 (4.3)	9 (5.7)
Self-reported autoimmune disease diagnosis, n (%)	9 (2.3)	8 (3.4)	1 (0.6)
Self-reported inflammatory bowel disease diagnosis, n (%)	5 (1.3)	4 (1.7)	1 (0.6)
PAPP-A2, ng/mL, median (P25, P75)	0.25 (0.19, 0.33)	0.26 (0.20, 0.33)	0.24 (0.18, 0.32)
PAPP-A, ng/mL (values above detection limit), median (P25, P75)	6.6 (4.8, 9.9)	5.9 (4.5, 8.5)	7.7 (5.4, 10.4)
PAPP-A, ng/mL (values below detection limit substituted), median (P25, P75)	5.1 (1.5, 8.2)	4.6 (1.5, 6.9)	6.5 (3.8, 9.9)
Stanniocalcin-2, ng/mL, median (P25, P75)	87.6 (72.8, 102.9)	86.7 (70.8, 103.4)	88.2 (74.7, 102.9)
Total IGF-1, ng/mL, median (P25, P75)	177.4 (138.4, 227.6)	168.8 (131.8, 214.8)	188.6 (145.7, 244.9)
free IGF-1, ng/mL, median (P25, P75)	1.02 (0.38, 2.07)	1.20 (0.48, 2.31)	0.78 (0.28, 1.71)
IGFBP-1, ng/mL, median (P25, P75)	1.98 (0.90, 3.90)	2.59 (1.19, 4.80)	1.39 (0.61, 2.75)
IGFBP-2, ng/mL, median (P25, P75)	302.0 (201.6, 437.0)	321.5 (203.9, 451.0)	282.4 (197.3, 406.1)
IGFBP-3, ng/mL, median (P25, P75)	3579 (3177, 3965)	3630 (3295, 4036)	3497 (3063, 3870)
IGFBP-5, ng/mL, median (P25, P75)	22.6 (6.2, 73.7)	21.3 (4.5, 65.2)	23.6 (7.9, 85.2)
free to total IGF-1 ratio, %, median (P25, P75)	0.54 (0.24, 1.13)	0.65 (0.30, 1.20)	0.42 (0.17, 0.88)
IGF1/IGFBP-3 molar ratio, %, median (P25, P75)	17.8 (14.4, 22.3)	16.8 (13.5, 20.6)	19.6 (16.3, 24.0)

N = 394, except for results on free IGF-1 (n = 339), IGFBP-1 (n = 360), IGFBP-5 (n = 125), and PAPP-A above detection limit (N = 288).

*BMI* body mass index, *PAPP-A* pregnancy-associated plasma protein A, *PAPP-A2* pregnancy-associated plasma protein A2, *STC2* stanniocalcin 2, *SD* standard deviation.

PAPP-A2 correlated positively, and STC2 inversely with age, while PAPP-A was not correlated with age (Table 2). Total and free IGF-1 as well as IGFBP-3 and the IGF-1 to IGFBP-3 molar ratio correlated inversely with age, while IGFBP-2 was positively correlated with age. In Pearson partial correlation analysis, controlling for age, sex, BMI and pretests, circulating PAPP-A was not statistically significantly correlated with total or free IGF-1 (Table 2). A positive correlation of PAPP-A was only observed for the IGF-1/IGFBP-3 molar ratio. In addition, PAPP-A was positively correlated with IGFBP-5, but not with IGFBP-2. PAPP-A2 was statistically significantly inversely correlated with total IGF-1, IGFBP-3 and the IGF-1:IGFBP-3 molar ratio, but not with free IGF-1 or the free to total IGF-1 ratio. PAPP-A2 was not significantly correlated with IGFPB-5, but statistically significantly positively correlated with IGFBP-2. The pappalysin-inhibitor STC2 was inversely correlated with total but not free IGF-1, positively correlated with IGFBP-1 and inversely correlated with IGFBP-2 and IGFBP-3. STC2 correlated statistically significantly positively with PAPP-A2 (r = 0.10, *p* = 0.05) but not with PAPP-A (r = − 0.08, *p* = 0.16 data not shown in Table 2).

**Table 2:** Pearson partial correlation coefficients ofPAPP-A, PAPP-A2 and STC with IGF-1, and IGF-binding proteins^*^.

	Age	Total IGF-1	Free IGF-1^†^	IGFBP-1^†^	IGFBP-2	IGFBP-3	IGFBP-5^†^	Free to total IGF-1 ratio^†^	IGF-1/IGFBP3 molar ratio
Age		**− 0.42**	**− 0.20**	0.005	**0.16**	**− 0.25**	− 0.08	− 0.10	**− 0.33**
		**(*p*** **< 0.001)**	**(*p* < 0.001)**	(*p* = 0.93)	**(*p* = 0.002)**	**(*p* < 0.001)**	(*p* = 0.39)	(*p* = 0.06)	**(*p* < 0.001)**
PAPP-A^†^ (values above detection limit)	− 0.04	0.09	0.10	0.06	0.03	− 0.04	**0.30**	0.08	**0.12**
	(*p* = 0.45)	(*p* = 0.12)	(*p* = 0.12)	(*p* = 0.32)	(*p* = 0.61)	(*p* = 0.52)	**(*p*** **= 0.004)**	(*p* = 0.21)	**(*p*** **= 0.04)**
PAPP-A^†^ (values below detection limit substituted)	0.03	0.06	0.08	0.04	0.10	− 0.02	**0.30**	0.07	0.07
	(*p* = 0.56)	(*p* = 0.24)	(*p* = 0.13)	(*p* = 0.43)	(*p* = 0.06)	(*p* = 0.76)	**(*p*** **= 0.0009)**	(*p* = 0.18)	(*p* = 0.16)
PAPP-A2	**0.19**	**− 0.16**	0.005	0.07	**0.17**	**− 0.10**	− 0.03	0.06	**− 0.13**
	**(*p* < 0.001)**	**(*p* = 0.002)**	(*p* = 0.93)	(*p* = 0.16)	**(*p* < 0.001)**	**(*p* < 0.05)**	(*p* = 0.78)	(*p* = 0.31)	**(*p* = 0.008)**
Stanniocalcin-2	**− 0.21**	**− 0.12**	0.04	**0.18**	**− 0.16**	**− 0.11**	− 0.05	0.07	− 0.06
	**(*p* < 0.001)**	**(*p* = 0.02)**	(* p* = 0.41)	**(*p* = 0.0005)**	**(*p* = 0.0016)**	**(*p* = 0.03)**	(*p* = 0.56)	(*p* = 0.18)	(*p* = 0.24)

N = 394, except for results on IGFBP-1 (n = 360), IGFBP-5 (n = 125), free IGF-1 (N = 339) and PAPP-A above detection limit (N = 288).

*Correlations between biomarkers were adjusted for age, sex, BMI, and pretest; correlations with age were adjusted for sex.

^†^IGFBP-1, IGFBP-5, free IGF-1, free-to-total IGF-1 ratio and PAPP-A were log-transformed for analysis.

Significant values are in bold.

Multivariable adjusted (for age, sex, BMI and pretests) mean concentrations of IGF-1 and its binding proteins across categories of PAPP-A and PAPP-A2 as well as continuous associations are shown in Tables 3 and 5. In accordance with the correlation analysis, we observed no association between PAPP-A and total or free IGF-1. However, a positive association was observed with the PAPP-A substrates IGFBP-2 and IGFBP-5, with statistically significant trends across PAPP-A categories (Table 3), although the association between IGFBP-2 was not statistically significant in the continuous analysis. The positive association between PAPP-A and IGFBP-5 was not changed by additional adjustment for IGF-1 or other IGFBPs (Table 4).

**Table 3:** Multivariable adjusted* plasma concentrations of IGF-1 and IGF-binding protein concentrations by categories of PAPP-A concentrations.

	PAPP-A					P-trend	β (95% CI) per log-transformed PAPP-A (values above detection limit)^§^	β (95% CI) per log-transformed PAPP-A (values below detection limit substituted))^§^
	Below detection limit	Quartile 1	Quartile 2	Quartile 3	Quartile 4			
IGF-1, ng/ml^†^	185.7	177.3	201.9	185.2	185.5		7.8	4.3
	(172.8, 198.6)	(162.6, 192.0)	(187.8, 216.1)	(170.8, 199.6)	(171.1, 200.0)	0.99	(− 2.0, 17.7)	(− 2.7, 11.2)
Free IGF1^‡§^ (values above detection limit)	0.80	0.83	0.97	0.67	1.08		1.20	1.12
	(0.58, 1.10)	(0.58, 1.18)	(0.69, 1.36)	(0.47, 0.96)	(0.77, 1.53)	0.24	(0.95, 1.51)	(0.96, 1.32)
IGFBP-1, ng/mL^‡║§^	2.00	1.61	2	2.26	2.28		1.12	1.06
	(1.54, 2.59)	(1.22, 2.12)	(1.53, 2.63)	(1.72, 2.96)	(1.73, 3.01)	0.16	(0.94, 1.32	(0.94, 1.19)
IGFBP-2, ng/mL^†^	302.6	321.3	319.4	338.2	384.1		6.7	16.6
	(270.1, 335.1)	(284.3, 358.3)	(283.8, 355.0)	(302.0, 374.4)	(347.8, 420.5)	**0.001**	(− 19.2, 32.7)	(− 0.9, 34.1)
IGFBP-3, ng/mL^†^	3537	3558	3707	3523	3464		− 32.0	− 9.2
	(3398, 3676)	(3400, 3717)	(3555, 3860)	(3368, 3678)	(3308, 3619)	0.28	(− 139.2, 75.2)	(− 83.6, 65.2)
IGFBP-5, ng/mL^‡§^	13.1	18.1	17.3	36.0	33.1		**1.97**	**1.69**
	(7.1, 24.3)	(7.8, 42.4)	(8.4, 35.7)	(17.8, 72.8)	(17.0, 64.2)	**0.02**	**(1.26, 3.12)**	**(1.25, 2.29)**
IGF-1/IGFBP-3 molar ratio, %^†^	19.0	18.0	19.9	19.0	19.4		0.92	0.44
	(17.9, 20.2)	(16.6, 19.3)	(18.7, 21.2)	(17.6, 20.3)	(18.1, 20.7)	0.54	(0.02, 1.82)	(− 0.19, 1.07)
Free to total IGF-1 ratio, %^‡§^	0.43	0.49	0.49	0.36	0.60		1.15	1.10
	(0.32, 0.59)	(0.35, 0.69)	(0.35, 0.69)	(0.26, 0.51)	(0.43, 0.84)	0.23	(0.92, 1.45)	(0.94, 1.29)
PAPP-A2, ng/mL^†^	0.27	0.27	0.27	0.28	0.28		0.01	0.01
	(0.24, 0.29)	(0.24, 0.29)	(0.25, 0.30)	(0.25, 0.30)	(0.25, 0.31)	0.25	(− 0.01, 0.03)	(− 0.01, 0.02)
Stanniocalcin-2, ng/mL^†^	92.3	88.7	85.7	80.7	82.0		− 2.5	**− 4.0**
	(86.6, 98.05)	(82.2, 95.3)	(79.4, 92.0)	(74.3, 87.1)	(75.6, 88.4)	**0.01**	(− 6.3, 1.0)	**(− 7.1, − 0.9)**

N = 394, except for results on free IGF-1 (N = 339), IGFBP-1 (N = 360), IGFBP-5 (N = 125), and PAPP-A above detection limit (N = 288).

*Adjusted for age, sex, BMI, and pretest.

^†^Results for IGF-1, IGFBP-2, IGFBP-3, IGF-1/IGFBP-3 molar ratio, PAPP-A2 and stanniocalcin-2 are means (95%-confidence intervals).

^‡^Results for IGFBP-1, IGFBP-5, free IGF-1 and free to total IGF-1 ratio are geometric means (95%-confidence intervals).

^║^Models for IGFBP-1 were additionally adjusted for fasting status (≥ 6 h, yes vs no).

^§^For IGFBP-1, IGFBP-5, free IGF-1, and free to total IGF-1 ratio  β estimates per log-transformed PAPP-A increment were back-transformed from the logarithmic to the original scale; back transformed estimates can be interpreted as x-fold IGFBP-1 or IGFBP-5 or free IGF-1 concentrations or free to total IGF-1 ratio associated with one unit higher log-transformed PAPP-A.

Significant values are in bold.

**Table 4:** Association of PAPP-A with IGFBP-5 with additional adjustment for IGF-1 or other IGFBPs.

	n	β per log-transformed PAPP-A (values above detection limit)	95% CI	β per log-transformed PAPP-A (values below detection limit substituted))	95% CI
IGFBP-5, ng/mL^‡^					
As in Table 3	125	**1.97**	**(1.26, 3.12)**	**1.69**	**(1.25, 2.29)**
- Plus adjustment for IGF-1	125	**1.98**	**(1.27, 3.10)**	**1.68**	**(1.24, 2.26)**
- Plus adjustment for IGFBP-2	125	**1.97**	**(1.25, 3.11)**	**1.66**	**(1.23, 2.26)**
- Plus adjustment for IGFBP-3	125	**1.99**	**(1.26, 3.15)**	**1.71**	**(1.26, 2.32)**
In participants with available IGFBP-1	116	**2.08**	**(1.31, 3.31)**	**1.97**	**(1.44, 2.69)**
- Plus adjustment for IGFBP-1 (log-transformed)	116	**2.08**	**(1.31, 3.31)**	**1.96**	**(1.44, 2.69)**
In participants with available free IGF-1	110	**1.87**	**(1.18, 2.95)**	**1.52**	**(1.11, 2.08)**
- Plus adjustment for free IGF-1 (log-transformed)	110	**1.83**	**(1.16, 2.91)**	**1.48**	**(1.08, 2.04)**
- Plus adjustment for free to total IGF-1 (%, log-transformed)	110	**1.85**	**(1.17, 2.95)**	**1.50**	(1.10, 2.07)

*adjusted for age, sex, BMI, and pretest and IGF-1/IGFBPs as indicated.

^**‡**^IGFBP-5 β estimates per log-transformed PAPP-A increment were back-transformed from the logarithmic to the original scale; back transformed estimates can be interpreted as x-fold IGFBP-5 concentrations associated with one unit higher log-transformed PAPP-A.

Significant values are in bold.

PAPP-A2 concentration was statistically significantly inversely associated with total IGF-1 concentrations as well as the IGF-1/IGFBP-3 molar ratio (both in the quintile and continuous models), but not associated with free IGF-1 or the percentage of free to total IGF-1 (Table 5). No significant association was observed between PAPP-A2 and concentrations of the PAPP-A2 substrates IGFBP-3 and IGFBP-5. PAPPA-2 was positively associated with IGFBP-2 concentration both in the quintile and the continuous model.

**Table 5:** Multivariable adjusted* plasma concentrations of IGF-1 and IGF-binding protein concentrations by age- and sex-specific quintiles and continuously per increment of PAPP-A2 concentrations.

	PAPP-A2					P-trend	β (95% CI) per 0.05 ng/mL^§^
	Quintile 1	Quintile 2	Quintile 3	Quintile 4	Quintile 5		
IGF-1, ng/ml^†^	189.9	198.2	204.9	167.7	178.1		**− 4.3**
	(175.8, 203.9)	(184.0, 212.3)	(191.7, 218.1)	(154.2, 181.1)	(164.5, 191.7)	**0.009**	**(− 7.0, − 1.6)**
Free IGF1 ^‡§^ (values above detection limit)	0.58	1.12	1.25	0.69	0.84		0.99
	(0.41, 0.83)	(0.79, 1.59)	(0.90, 1.73)	(0.49, 0.95)	(0.61, 1.16)	0.75	(0.93, 1.06)
IGFBP-1, ng/mL^‡║§^	1.74	1.79	2.09	2.52	2.04		1.04
	(1.32, 2.30)	(1.37, 2.35)	(1.60, 2.72)	(1.91, 3.31)	(1.57, 2.64)	0.179	(0.99, 1.09)
IGFBP-2, ng/mL^†^	295.6	319.3	309.3	343.7	384.4		**11.9**
	(259.7, 331.5)	(283.1, 355.5)	(275.6, 343.1)	(309.3, 378.1)	(349.6, 419.2)	**0.0002**	**(5.0, 18.8)**
IGFBP-3, ng/mL^†^	3600	3594	3699	3370	3532		− 27.8
	(3447, 3753)	(3439, 3748)	(3556, 3843)	(3224, 3517)	(3383, 3680)	0.15	(− 57.3, 1.8)
IGFBP-5, ng/mL^‡§^	15.0	35.4	19.5	23.1	21.2		0.97
	(7.3, 30.9)	(16.8, 74.4)	(10.1, 37.7)	(11.5, 46.7)	(10.2, 44.2)	0.98	(0.85, 1.11
IGF-1/IGFBP-3 molar ratio, %^†^	19.2	19.8	20.2	18.0	18.3		**− 0.34**
	(17.9, 20.5)	(18.5, 21.1)	(19.0, 21.5)	(16.8, 19.2)	(17.0, 19.6)	**0.04**	**(− 0.59, − 0.09)**
Free to total IGF-1 ratio, %^‡§^	0.31	0.57	0.63	0.41	0.50		1.03
	(0.22, 0.44)	(0.40, 0.80)	(0.46, 0.87)	(0.30, 0.56)	(0.36, 0.68)	0.30	(0.97, 1.10)
PAPP-A, ng/mL ^‡§^^†^(values above detection limit)	6.10	7.84	7.60	7.49	7.07		1.01
	(4.99, 7.46)	(6.38, 9.63)	(6.36, 9.09)	(6.19, 9.06)	(5.90, 8.47)	0.57	(0.98, 1.05)
PAPP-A, ng/mL^‡^^§^(values below detection limit substituted)	4.44	4.99	5.67	4.97	5.42		1.02
	(3.61, 5.47)	(4.04, 6.15)	(4.66, 6.89)	(4.07, 6.07)	(4.43, 6.63)	0.37	(0.98, 1.06)
Stanniocalcin-2, ng/mL^†^	80.1	84.8	85.4	90.0	88.8		1.4
	(73.7, 86.5)	(78.3, 91.3)	(79.4, 91.4)	(83.8, 96.1)	(82.6, 95.0)	0.03	(0.2, 2.6)

N = 394, except for results on free IGF-1 (N = 339), IGFBP-1 (N = 360), IGFBP-5 (N = 125), and PAPP-A above detection limit (N = 288).

*Adjusted for age, sex, BMI, and pretest.

^†^Results for IGF-1, IGFBP-2, IGFBP-3, IGF-1:IGFBP-3 molar ratio and stanniocalcin-2 are means (95%-confidence intervals).

^‡^Results for IGFBP-1, IGFBP-5, free IGF-1, free to total IGF-1 ratio, and PAPP-A are geometric means (95%-confidence intervals).

^║^Models for IGFBP-1 were additionally adjusted for fasting status (≥ 6 h, yes vs no).

^§^For IGFBP-1, IGFBP-5, free IGF-1, free to total IGF-1 ratio and PAPP-A β estimates per 0.5 ng/mL PAPP-A2 increment were back-transformed from the logarithmic to the original scale; back transformed estimates can be interpreted as x-fold IGFBP-1, IGFBP-5, free IGF-1 or PAPP-A concentrations or free to total IGF-1 ratio associated with 0.5 ng/mL higher PAPP-A2.

Significant values are in bold.

The inverse association of PAPP-A2 with total IGF-1 was only weakly attenuated and remained statistically significant after adding free IGF-1 or IGFBPs one by one to the model (Table 6). In the subsample with available IGFBP-5 measurement (n = 125) no significant association of PAPP-A2 with total IGF-1 was observed with or without adjustment for IGFBP-5, and adjustment for IGFBP-5 only marginally affected the beta estimate. The inverse association of PAPP-A2 with the IGF-1:IGFBP-3 molar ratio persisted after additional adjustment for free IGF-1, IGFBP-2 or IGFBP-1. The positive association of PAPP-A2 with IGFBP-2 was only weakly attenuated and remained statistically significant after additional adjustment for free or total IGF-1, IGFBP-3, or IGFBP-1.

**Table 6:** Associations of PAPP-A2 with IGF-1, IGF-1:IGFBP-3 molar ratio and IGFBP-2 with additional adjustment for other members of the system.

	n	β per 0.05 ng/mL PAPP-A2 increment	95% CI
IGF-1, ng/ml			
As in Table 5	394	**− 4.3**	**(− 7.0, − 1.6)**
- Plus adjustment for IGFBP-2	394	**− 3.8**	**(− 6.5, − 1.0)**
- Plus adjustment for IGFBP-3	394	**− 3.1**	**(− 5.6, − 0.7)**
In participants with available IGFBP-1	360	**4.0**	**(− 6.8, − 1.2)**
- Plus adjustment for IGFBP-1 (log-transformed)	360	**− 3.3**	**(− 5.9, − 0.6)**
In participants with available IGFBP-5	125	− 2.6	(− 7.5, 2.2)
- Plus adjustment for IGFBP-5 (log-transformed)	125	− 2.4	(− 7.2, 2.4)
In participants with available free IGF-1	125	**− 4.5**	**(− 7.4, − 1.6)**
- Plus adjustment for free IGF-1 (log-transformed)	339	**− 4.3**	**(− 7.2, − 1.3)**
IGF-1/IGFBP-3 molar ratio, %			
As in Table 5	394	**- 0.34**	(− **0.59**, − **0.09**)
- Plus adjustment for IGFBP-2	394	**− 0.35**	**(− 0.60, − 0.09)**
In participants with available IGFBP-1	360	**- 0.33**	**(− 0.58, − 0.08)**
- Plus adjustment for IGFBP-1 (log-transformed)	360	**− 0.29**	**(− 0.53, − 0.04)**
In participants with available IGFBP-5	125	- 0.13	(− 0.58, 0.32)
- Plus adjustment for IGFBP-5 (log-transformed)	125	− 0.11	(− 0.55, 0.33)
In participants with available free IGF-1	339	**− 0.37**	**(− 0.64, − 0.09)**
- Plus adjustment for free IGF-1 (log-transformed)	339	**− 0.37**	**(− 0.64, − 0.10)**
IGFBP-2, ng/ml			
As in Table 5	394	**11.9**	**(5.0, 18.8)**
- Plus adjustment for IGFBP-3	394	**10.3**	**(3.6, 17.1)**
- Plus adjustment for IGF-1	394	**10.7**	**(3.7, 17.6)**
In participants with available free IGF-1	339	**11.1**	**(4.0, 18.2)**
- Plus adjustment for free IGF-1 (log-transformed)	339	**11.1**	**(4.1, 18.1)**
- Plus adjustment for free to total IGF-1 (%, log-transformed)	339	**11.5**	**(4.4, 18.5)**
In participants with available IGFBP-1	360	**10.9**	**(3.7, 18.0)**
- Plus adjustment for IGFBP-1 (log-transformed)	360	**9.2**	**(2.3, 16.2)**
In participants with available IGFBP-5	125	4.9	(− 7.1, 17.0)
- Plus adjustment for IGFBP5 (log-transformed)	125	5.2	(− 6.9, 17.2)

Model 1: adjusted for age, sex, BMI, and pretest and IGF-1/IGFBPs as indicated.

Significant values are in bold.

Multivariable adjusted mean concentrations of IGF-1, IGFBPs and PAPP-A/PAPP-A2 across STC2 quintiles are shown in Table 7. STC2 concentration was statistically significantly inversely associated with total (but not free) IGF-1 as well as IGFBP-2 and IGFBP-3. In addition, STC2 was statistically significantly positively associated with IGFBP-1 (multivariable adjusted model including fasting status). Weak inverse associations between STC2 and PAPP-A2 and PAPP-A were also observed. The inverse association between STC2 and IGF-1 was attenuated after additional adjustment for PAPP-A and PAPP-A2 as well as after additional adjustment for free IGF-1 and IGFBPs (Table 8). The positive association of STC2 with IGFBP-1 and the inverse association with IGFBP-2 persisted after additional adjustment for other IGFBPs or IGF-1, and also after adjustment for PAPP-A and PAPP-A2. The inverse association of STC2 with IGFBP-3 was attenuated after additional adjustment for IGF-1 or other IGFBPs.

**Table 7:** Multivariable adjusted* plasma concentrations of IGF-1 and IGF-binding protein concentrations by age- and sex-specific quintiles and continuously per increment of STC2 concentrations.

	STC2					P-trend	β (95% CI) per 10 ng/mL^§^
	Quintile 1	Quintile 2	Quintile 3	Quintile 4	Quintile 5		
IGF-1, ng/ml^†^	187.0	188.8	186.6	194.8	179.1		**− 2.4**
	(173.2, 200.8)	(174.8, 202.8)	(172.4, 200.7)	(180.6, 209.0)	(164.4, 193.8)	0.32	**(− 4.7, − 0.2)**
Free IGF1^‡^^§^ (values above detection limit)	0.64	0.84	0.95	1.16	0.88		1.03
	(0.45, 0.90)	(0.61, 1.16)	(0.67, 1.34)	(0.83, 1.62)	(0.61, 1.27)	0.26	(0.97, 1.09)
IGFBP-1, ng/mL^‡║§^	2.00	1.70	2.00	1.90	2.62		**1.07**
	(1.54, 2.60)	(1.30, 2.23)	(1.52, 2.64)	(1.46, 2.48)	(1.98, 3.47)	**0.02**	**(1.03, 1.10)**
IGFBP-2, ng/mL^†^	345.7	368.7	322.2	317.4	296.8		**− 8.9**
	(310.9, 380.5)	(333.4, 404.0)	(286.6, 357.9)	(281.7, 353.1)	(259.9, 333.8)	**0.01**	**(− 14.5, − 3.3)**
IGFBP-3, ng/mL^†^	3621	3543	3533	3607	3458		**− 24.9**
	(3472, 3770)	(3392, 3694)	(3380, 3685)	(3455, 3760)	(3300, 3616)	0.23	**(− 48.8, − 1.0)**
IGFBP-5, ng/mL^‡§^	25.2	12.3	28.5	30.1	17.1		0.96
	(12.9, 49.3)	(6.3, 24.2)	(14.7, 55.3)	(13.6, 66.6)	(7.6, 38.5)	0.97	(0.86, 1.08)
IGF-1/IGFBP-3 molar ratio, %^†^	18.7	19.3	19.3	19.4	18.9		− 0.09
	(17.4, 20.0)	(18.0, 20.6)	(18.0, 20.6)	(18.1, 20.7)	(17.5, 20.2)	0.79	(− 0.29, 0.11)
Free to total IGF-1 ratio, %^‡^^§^	0.35	0.47	0.51	0.6	0.51		1.04
	(0.25, 0.49)	(0.34, 0.65)	(0.37, 0.72)	(0.43, 0.83)	(0.36, 0.73)	0.15	(0.99, 1.10)
PAPP-A2, ng/mL^†﻿^	0.25	0.27	0.26	0.29	0.30		0.00
	(0.22, 0.27)	(0.25, 0.30)	(0.24, 0.29)	(0.27, 0.32)	(0.28, 0.33)	**0.01**	(0.00, 0.01)
PAPP-A, ng/mL^‡^^§^ (values above detection limit)	8.26	7.50	6.73	6.10	7.18		0.97
	(6.90, 9.89)	(6.20, 9.06)	(5.53, 8.20)	(5.06, 7.35)	(5.80, 8.89)	0.16	(0.94, 1.01)
PAPP-A, ng/mL^‡^^§^ (values below detection limit substituted)	6.08	5.31	4.56	4.86	4.54		**0.95**
	(4.98, 7.42)	(4.34, 6.50)	(3.71, 5.59)	(3.96, 5.96)	(3.68, 5.62)	**0.02**	**(0.93, 0.99)**

N = 394, except for results on free IGF-1 (N = 339), IGFBP-1 (N = 360), IGFBP-5 (N = 125), and PAPP-A above detection limit (N = 288).

*Adjusted for age, sex, BMI, and pretest.

^†^Results for IGF-1, IGFBP-2, IGFBP-3, IGF-1/IGFBP-3 molar ratio and PAPP-A2 are means (95%-confidence intervals).

^‡^Results for IGFBP-1, IGFBP-5, free IGF-1, free to total IGF-1 ratio and PAPP-A are geometric means (95%-confidence intervals).

^║^Models for IGFBP-1 were additionally adjusted for fasting status (≥ 6 h, yes vs no).

^§^IGFBP-1, IGFBP-5, free IGF-1, free to total IGF-1 ratio and PAPP-A estimates per 10 ng/mL STC2 increment on a logarithmic scale were back-tranformed.

Significant values are in bold.

**Table 8:** Associations of IGF-1 and IGF-binding proteins with STC2, mutually adjusted for other members of the system members*.

	n	β (95% CI) per 10 ng/mL^§^	95% CI
IGF-1, ng/ml			
As in Table 7	394	**− 2.4**	(− 4.7, − 0.2)
- Plus adjustment for PAPP-A2	394	− 2.1	(− 4.3, 0.1)
- Plus adjustment for PAPP-A (< LOD substituted, log-transformed)	394	**− 2.3**	**(− 4.5, − 0.1)**
- Plus adjustment for PAPP-A (< LOD substituted, log-transformed) and PAPP-A2	394	− 1.9	(− 4.1, 0.3)
- Plus adjustment for IGFBP-2	394	**− 3.0**	**(− 5.2, − 0.8)**
- Plus adjustment for IGFBP-3	394	− 1.4	(− 3.4, 0.6)
In participants with available IGFBP-1	360	− 2.1	(− 4.4, 0.2)
- Plus adjustment for IGFBP-1 (log-transformed)	360	− 0.9	(− 3.1, 1.3)
In participants with available IGFBP-5	125	− 2.0	(− 6.0, 2.2)
- Plus adjustment for IGFBP-5 (log-transformed)	125	− 1.7	(− 5.7, 2.3)
In participants with available free IGF-1	339	− 2.0	(− 4.5, 0.5)
- Plus adjustment for free IGF-1 (log-transformed)	339	− 2.3	(− 4.8, 0.1)
IGFBP-1, ng/mL^‡║^			
As in Table 7	360	**1.07**	**(1.03, 1.11)**
- Plus adjustment for PAPP-A2	360	**1.06**	**(1.03, 1.11)**
- Plus adjustment for PAPP-A (< LOD substituted, log-transformed)	360	**1.07**	**(1.03, 1.11)**
- Plus adjustment for PAPP-A (< LOD substituted, log-transformed) and PAPP-A2	360	**1.07**	**(1.03, 1.10)**
- Plus adjustment for IGF-1	360	**1.06**	**(1.02, 1.10)**
- Plus adjustment for IGFBP-2	360	**1.09**	**(1.05, 1.13)**
In participants with available IGFBP-5	116	**1.09**	**(1.02, 1.16)**
- Plus adjustment for IGFBP-5 (log-transformed)	116	**1.09**	**(1.02, 1.16)**
In participants with available free IGF-1	309	**1.04**	**(1.00, 1.08)**
- Plus adjustment for free IGF-1 (log-transformed)	309	**1.04**	**(1.00, 1.09)**
- Plus adjustment for free to total IGF-1 (%, log-transformed)	309	**1.04**	**(1.00, 1.09)**
IGFBP-2, ng/ml			
As in Table 7	394	**− 8.9**	**(− 14.5, − 3.3)**
- Plus adjustment for PAPP-A2	394	**− 10.2**	**(− 15.7, − 4.6)**
- Plus adjustment for PAPP-A (< LOD substituted, log-transformed)	394	**− 8.4**	**(− 14.0, − 2.7)**
- Plus adjustment for PAPP-A (< LOD substituted, log-transformed) and PAPP-A2	394	**− 9.7**	**(− 15.3, − 4.1)**
- Plus adjustment for IGF-1	394	**− 9.9**	**(− 15.5, − 4.3)**
- Plus adjustment for IGFBP-3	394	**− 10.5**	**(− 16.0, − 5.1)**
In participants with available free IGF-1	339	**− 13.9**	**(− 19.9, − 8.0)**
- Plus adjustment for free IGF-1 (log-transformed)	339	**− 13.5**	**(− 19.5, − 7.6)**
- Plus adjustment for free to total IGF-1 (%, log-transformed)	339	**− 13.6**	**(− 19.5, − 7.6)**
In participants with available IGFBP-1	360	**− 9.6**	**(− 15.4, − 3.7)**
- Plus adjustment for IGFBP-1 (log-transformed)	360	**− 12.7**	**(− 18.4, − 7.1)**
In participants with available IGFBP-5	125	**− 13.6**	**(− 23.4, − 3.7)**
- Plus adjustment for IGFBP-5 (log-transformed)	125	**− 13.3**	**(− 23.1, − 3.4)**
IGFBP-3, ng/ml			
As in Table 7	394	**− 24.9**	**(− 48.8, − 1.0)**
- Plus adjustment for PAPP-A2	394	− 22.6	(− 46.6, 1.4)
- Plus adjustment for PAPP-A (< LOD substituted, log-transformed)	394	**− 25.7**	**(− 49.8, − 1.5)**
- Plus adjustment for PAPP-A (< LOD substituted, log-transformed) and PAPP-A2	394	− 23.3	(− 47.5, 1.0)
- Plus adjustment for IGF-1	394	− 12.8	(− 34.1, 8.6)
- Plus adjustment for IGFBP-2	394	**− 35.4**	**(− 58.7, − 12.2)**
In participants with available free IGF-1	339	− 5.5	(− 31.2, 20.2)
- Plus adjustment for free IGF-1 (log-transformed)	339	− 7.0	(− 32.6, 18.6)
- Plus adjustment for free to total IGF-1 (%, log-transformed)	339	− 5.6	(− 31.4, 20.3)
In participants with available IGFBP-1	360	**− 24.9**	**(− 49.6, − 0.2)**
- Plus adjustment for IGFBP-1 (log-transformed)	360	− 15.8	(− 40.3, 8.8)
In participants with available IGFBP-5	125	- 6.3	(− 46.7, 34.1)
- Plus adjustment for IGFBP-5 (log-transformed)	125	− 6.0	(− 46.6, 34.7)

*Adjusted for age, sex, BMI, and pretest and IGF-1/IGFBPs/PAPP-A/PAPP-A2 as indicated.

^║^Models for IGFBP-1 were additionally adjusted for fasting status (≥ 6 h, yes vs no).

^**‡**^IGFBP-1 β estimates per 10 ng/mL STC2 increment were back-transformed from the logarithmic to the original scale; back transformed estimates can be interpreted as x-fold IGFBP-1 concentrations associated with 10 ng/mL higher STC2.

Significant values are in bold.

When we excluded participants with self-reported prevalent diseases, multivariable adjusted estimates for the association between PAPP-A, PAPP-A2 or STC2 and IGF-1 and IGF-binding proteins were not appreciably altered (Supplemental Table S1).

## Discussion

Here we sought to investigate the association of the circulating pappalysins PAPP-A and PAPP-A2 and the pappalysin inhibitor STC2 with IGF-1 and its binding proteins across a general adult human population with a wide age range. Given the proteolytic activities of PAPP-A and PAPP-A2 towards IGFBPs and because the half-life of IGF-1 is reduced when it is not bound to IGFBPs, we hypothesized that higher circulating PAPP-A or PAPP-A2 would be associated with lower total IGF-1 concentrations and with higher free IGF-1. In line with our hypothesis, we observed inverse associations of PAPP-A2 with IGF-1 as well as with the IGF-1:IGFBP-3 molar ratio, but no associations with free IGF-1 or the percentage of free to total IGF-1. A positive association was observed between PAPP-A2 and IGFBP-2. Interestingly, the positive association between PAPP-A2 and IGFBP-2 as well as the inverse associations with IGF-1 and the IGF-1:IGFBP-3 molar ratio persisted after adjustment for various members of the system, suggesting that circulating concentrations of these members (some of which may be considered as intermediary variables based on their effect observed in experimental studies) do not statistically account for this association. We observed a positive correlation between PAPP-A2 and its substrate IGFBP-3 but no significant association in the multivariable model and no association between PAPP-A2 and its substrate IGFBP-5. With respect to PAPP-A, no associations with free or total IGF-1 were observed, but a positive association with its substrate IGFBP-5 was observed. We also observed inverse associations between circulating STC2 and total IGF-1, IGFBP-2 and IGFBP-3, and a positive association with IGFBP-1, of which only the association with IGFBP-1 and IGFBP-2 persisted after adjustment for IGF-1, other IGFBPs or PAPP-A/PAPP-A2. The additional adjustment for PAPP-A and PAPP-A2 suggests that the association of STC2 with IGF-1 and IGFBP-3 is partly accounted for by circulating pappalysins (in the case of IGF-1 particularly by PAPP-A2), whereas the association between STC2 and IGFBP-1 and IGFBP-2 appear to be independent of circulating pappalysins. Our results suggest that both PAPP-A2 and STC2 play a role for IGF-1 metabolism, which may have important health implications in adults.

We are not aware of any observational studies in adult general population samples with a wide age range to compare our results to. In a cross-sectional study of 838 children aged 3–18 years old in the US, circulating PAPP-A2 was associated with lower free and total IGF-1, higher percentage of free to total IGF-1, and lower intact as well as higher total IGFBP-3^36^. In a more recent Spanish study, associations between PAPP-A and PAPP-A2 with members of the IGF-system and with STCs were investigated among newborns (n = 150 full-term, n = 40 preterm) and healthy individuals aged 1–30 years (n = 1071)^25^. The authors of that study observed an inverse association between PAPP-A2 and free or total IGF-1, and a positive association with the percentage of free to total IGF-1. In addition, they observed inverse correlations between PAPP-A2 and intact (but not total) IGFBP-3 as well as IGFBP-5 and no association with IGFBP-2.

PAPP-A2 does not show proteolytic activation towards other IGFBPs except IGFBP-3 and IGFBP-5^23,26^. However, we observed a positive association between PAPP-A2 and IGFBP-2 concentrations, to our knowledge for the first time in an adult human population and this association persisted after adjusting for other IGFBPs or IGF-1. Since IGFBP-2 is the second most abundant IGFBP in the circulation, concurrent changes in the IGF-1 system may be one possible explanation: in the presence of higher PAPP-A2 concentration (resulting in increased release of IGF-1 from its ternary complexes due to specific cleavage of IGFBP-3 or IGFBP-5), IGFBP-2 could possibly bind to the released (free) IGF-1 from the ternary complexes and build binary complexes, leading to a longer half-life of IGFBP-2 due to protection against the rapid elimination in the circulation. Rare homozygous mutations in the *PAPP-A2* gene resulting in loss-of-function of PAPP-A2 in humans have been described in young individuals (age between 3 and 22 years) from two families^30^. Within these families, affected individuals had higher circulating total IGF-1, IGFBP-3, IGFBP-5, ALS and IGF-2 levels, less free or bioactive IGF-1 as well as elevated GH secretion compared with their non-affected siblings. In animal models, *PAPP-A2* knockout mice presented higher total IGF-1, lower free IGF-1, lower IGFBP-5 and variable IGFBP-3 concentrations compared with wild-type mice^22^. Thus, our observations that higher PAPP-A2 concentrations are associated with lower total IGF-1 and IGFBP-3 concentrations are in line with observations on rare *PAPPA2* mutations leading to PAPP-A2 dysfunction in young individuals as well as with observations in animal models (*PAPPA2* knockout mice), except that we observed no association between PAPP-A2 and free IGF-1. Whether the lack of association between PAPP-A2 and free IGF-1 is related to the higher age in our study population compared with the two previous studies in children, adolescents and young adults where positive associations between PAPP-A2 and free IGF-1 were observed^36,46^, deserves further investigation.

We observed no associations between PAPP-A and circulating IGF-1 (total, free or percentage of free to total IGF-1), largely in line with the Spanish study in newborns and healthy individuals aged 1–30 years, where PAPP-A was not associated with total IGF-1 or the percentage of free to total IGF-1 but correlated positively with free IGF-1^25^. The lack of association between PAPP-A and IGF-1 could be explained by PAPP-A acting primarily at the cellular level, i.e. close to the IGF-1 receptor^35^, and therefore differences in circulating PAPP-A show less associations with circulating IGF-1.

The here observed inverse association of STC2 with IGFBP-2 and the positive association of STC2 with total IGF-1 are in line with the Spanish study in newborns and young people, although no correlation with IGFBP-3 was observed in their study (IGFBP-1 was not measured)^25^. We are not aware of other investigations in adults from the general population where STC2 was investigated in relation to IGF-1, its binding proteins or pappalysins.

Strengths of our study include the population-based study design with a wide age range, i.e. a sample of participants from the general population mainly drawn from population registries, highly standardized study procedures including the handling of blood samples^31,47^ as well as the use of standardized and validated (ELISA) immunoassays. The cross-sectional design and observational nature of our study limits the drawing of causal inferences, although experimental studies on that topic in humans are difficult to conduct for ethical reasons. We adjusted for potentially confounding factors including age, sex and BMI and pretest phase, but we cannot exclude that observed associations were due to residual confounding, e.g. by age, which correlates inversely with IGF-1^48^ and positively with PAPP-A2^31^. Our general population sample consisted of mainly healthy individuals, but certain chronic diseases were present to a certain extent (e.g. self-reported osteoporosis in 4%, diabetes in 5%, autoimmune disease in 2% and inflammatory bowel disease in 1% of participants)^31^. Because we aimed at an investigation in a general population sample, we did not exclude participants with prevalent diseases for our main analysis. However, exclusion of participants with self-reported prevalent diseases (including inflammatory bowel diseases that had been associated with higher PAPP-A2 concentrations in our previous study^31^) from our analysis did not change results appreciably. We cannot exclude that frozen storage (at − 80 °C) of blood samples for several years could have affected measurements. It has been shown that long-term storage of up to 22 years does not influence measurements of IGF-1, IGFBP-1 and IGFBP-3^49–51^. We are not aware of any published investigations on the effect of long-term storage on measurements of PAPP-A, PAPP-A2 and STC2. PAPP-A and PAPP-A2 can be considered as quite stable, even at − 20 °C or at room temperature and not sensitive to freeze/thaw cycles (Claus Oxvig, personal communication). It is a further limitation of our study that the ELISA assays used for IGFBP-2, IGFBP-3 and IGFBP-5 could not distinguish between the intact proteins, the IGF-bound proteins, and the fragments generated by proteolysis. In addition, assays from different manufacturers were used to determine total and free IGF-1. It is another limitation that we could not analyze IGFBP-4, the primary substrate of PAPP-A, because no easily applicable commercial assays were available. We encountered some proportions of values below the detection limit for our measurements of IGFBP-1, IGFBP-5, free IGF-1 and PAPP-A. In the case of PAPP-A, which was an independent variable in our models, we explored different ways of handling values below the detection limit, i.e. analyzing only those above the detection limit as well as replacing those below the detection limit with values of half the lower detection limit. Proportions with values below the detection limit have been observed in previous epidemiological studies investigating free IGF-1^52^ and IGFBP-1^53^ as independent variables, and similar approaches (assigning values of half the lower limit of detection) have been applied. Finally, participants were generally non-fasting (i.e. the majority of participants had eaten in the last 6 h before the blood draw), which is a limitation of our study, since fasting status has been shown to influence IGFBP-1 concentrations^45^. We therefore adjusted the multivariable models investigating IGFBP-1 for fasting status. Other biochemical molecules of the GH-IGF axis that might have affected our results were not available for the present analysis: GH upregulates IGF-1, IGFBP-3 and ALS^19^; circulating insulin up-regulates hepatic IGF-1 synthesis through actions on GH on the one hand, and suppresses hepatic secretion of IGFBP-1 and -2 on the other hand, thereby enhancing IGF-1 bioactivity^54,55^; ALS abundance also plays a role in the IGF system as it is an integral part of the ternary complexes^19^; IGF-2 measurement might have been informative since IGFBP-2 and -5 exhibit a greater affinity for IGF-2 than IGF-1^56^, but we here focused on IGF-1 due to its physio-pathological relevance. Moreover, other circulating molecules and metabolites may have an impact on the association between IGF-1 and PAPP-A2. For example, vitamin D has been shown to increase circulating IGF-1 concentrations in a small intervention study in adults^57^, but was not available for the present analysis. We found in our analyses that some of the associations remained statistically significant even after adjustment for potentially intermediary variables, such as the observed inverse association of PAPP-A2 with IGF-1 and the IGF-1:IGFBP-3 molar ratio, which remained significant after adjustment for other IGFBPs. While these observations may be considered as a hint that pathways other than the intermediary variables may be relevant one has to keep in mind that our analysis is based on circulating blood concentrations of proteins, which may not directly reflect pathways that are ongoing on a tissue level. Therefore, our findings do not contradict what has been observed in experimental studies.

In conclusion, after adjusting for age, sex, BMI, and pretest phase we observed an inverse association of plasma concentrations of PAPP-A2 with plasma concentrations of total IGF-1 as well as with the IGF-1:IGFBP-3 molar ratio, but not with free IGF-1 and a positive association with IGFBP-2, while circulating PAPP-A was not associated with IGF-1. The pappalysin inhibitor STC2 was inversely associated with total but not free IGF-1. Our findings support the hypothesis that PAPP-A2 as well as STC2 play a role for IGF-1 in adult humans. The potential role of PAPP-A2 and STC2 for health and disease, e.g. regarding the risk of cancer and metabolic diseases in adults warrants further investigation.

### Supplementary Information

Supplementary Table S1.

## Data Availability

The data that support the findings of this study are available from the NAKO (www.nako.de) but restrictions apply to the availability of these data, which were used under license for the current study, and so are not publicly available. Data are however available from the authors (contact: Tobias Pischon, tobias.pischon@mdc-berlin.de) upon reasonable request and with permission of the NAKO.
