# Supplementary material for: Pregnancy associated plasma protein-A2 (PAPP-A2) and stanniocalcin-2 (STC2) but not PAPP-A are associated with circulating total IGF-1 in a human adult population
Source: Sci Rep. 2024 Jan 20;14:1770. doi: 10.1038/s41598-024-52074-8 (PMC10799854; doi:10.1038/s41598-024-52074-8)
Supplement: Supplementary file 1 — Supplementary Table S1. [file 41598_2024_52074_MOESM1_ESM.docx]

**Table S1**: Multivariable adjusted* association between PAPP-A, PAPP-A2 or STC2 and IGF-1 and IGF-binding proteins in the full sample versus a sample with people with prevalant diseases (n=43) excluded

|  | PAPP-A | | | |  | PAPP-A2 | |  | STC2 | |
| --- | --- | --- | --- | --- | --- | --- | --- | --- | --- | --- |
|  | Full sample | Diseases excluded | Full sample | Diseases excluded |  | Full sample | Diseases excluded |  | Full sample | Diseases excluded |
|  | β (95% CI) per log-transformed PAPP-A (values above detection limit) | β (95% CI) per log-transformed PAPP-A (values above detection limit) | β (95% CI) per log-transformed PAPP-A (values below detection limit substituted)) | β (95% CI) per log-transformed PAPP-A (values below detection limit substituted)) |  | β (95% CI) per 0.05 g/mL^§^ | β (95% CI) per 0.05 g/mL^§^ |  | β (95% CI) per 10 ng/mL^§^ | β (95% CI) per 10 ng/mL^§^ |
| IGF-1, ng/ml^†^ | 7.8 | 9.0 | 4.3 | 5.2 |  | **-4.3** | **-4.9** |  | **-2.4** | **-2.5** |
|  | (-2.0, 17.7) | (-1.1, 19.1) | (-2.7, 11.2) | (-2.1, 12.5) |  | **(-7.0, -1.6)** | **(-7.9, -1.9)** |  | **(-4.7, -0.2)** | **(-4.9, -0.2)** |
| free IGF1 † (values above detection limit) | 1.20 | 1.15 | 1.12 | 1.13 |  | 0.99 | 0.99 |  | 1.03 | 1.02 |
|  | (0.95, 1.51) | (0.92, 1.46) | (0.96, 1.32 | (0.96, 1.33) |  | (0.93, 1.06) | (0.924, 1.06) |  | (0.97, 1.09) | (0.97, 1.09) |
| IGFBP-1, ng/mL^‡║^ | 1.12 | 1.12 | 1.06 | 1.05 |  | 1.04 | 1.04 |  | **1.07** | **1.06** |
|  | (0.94, 1.32) | (0.95, 1.3) | (0.94, 1.19) | (0.93, 1.2) |  | (0.99, 1.09) | (0.99, 1.10) |  | **(1.03, 1.10)** | **(1.02, 1.10)** |
| IGFBP-2, ng/mL^†^ | 6.7 | 5.6 | 16.6 | 17.5 |  | **11.9** | **13.9** |  | **-8.9** | **-9.1** |
|  | (-19.2, 32.7) | (-21.4, 32.7) | (-0.9, 34.1) | (-1.1, 36.1) |  | **(5.0, 18.8)** | **(6.2, 21.5)** |  | **(-14.5, -3.3)** | **(-15.1, -3.1)** |
| IGFBP-3, ng/mL^†^ | -32.0 | -46.2 | -9.2 | -29.1 |  | -27.8 | -32.0 |  | **-24.9** | **-30.0** |
|  | (-139.2, 75.2) | (-154.2, 61.76) | (-83.6, 65.2) | (-105.6, 47.32) |  | (-57.3, 1.8) | (-63.7, -0.2) |  | **(-48.8, -1.0)** | **(-54.8, -5.3)** |
| IGFBP-5, ng/mL^‡^ | **1.97** | **1.95** | **1.69** | **1.71** |  | 0.97 | 0.95 |  | 0.96 | 0.98 |
|  | **(1.26, 3.12)** | **(1.23, 3.10)** | **(1.25, 2.29)** | **(1.23, 2.37)** |  | (0.85, 1.11) | (0.82, 1.11) |  | (0.86, 1.08) | (0.87, 1.12) |
| IGF-1/IGFBP-3 molar ratio^†^ | 0.92 | 1.1 | 0.44 | 0.6 |  | **-0.34** | **-0.36** |  | -0.09 | -0.07 |
|  | (0.02, 1.82) | (0.20, 2.04) | (-0.19, 1.07) | (-0.04, 1.27) |  | **(-0.59, -0.09)** | **(-0.63, -0.09)** |  | (-0.29, 0.11) | (-0.29, 0.14) |
| free to total IGF-1 ratio, %^†^ | 1.15 | 1.10 | 1.10 | 1.10 |  | 1.03 | 1.03 |  | 1.04 | 1.04 |
|  | (0.92, 1.45) | (0.88, 1.38) | (0.94, 1.29) | (0.94, 1.29) |  | (0.97, 1.10) | (0.96, 1.10) |  | (0.99, 1.10) | (0.98, 1.10) |

Full sample: N=394, except for results on free IGF-1 (N=339), IGFBP-1 (N=360) and for results for IGFBP-5 (N=125)

Sample with exclusion of n=43 prevalent diseases (self-reported physician diagnosis of osteoporosis, diabetes, autoimmune or chronic inflammatory bowel disease): N=351, except for results on free IGF-1 (N=302), IGFBP-1 (N=320) and for results for IGFBP-5 (N=115)

*adjusted for age, sex, BMI, and pretest

^║^ models for IGFBP-1 were additionally adjusted for fasting status (≥ 6 hours, yes vs no)

^†^results for IGF-1, IGFBP-2, IGFBP-3 and IGF-1/IGFBP-3 molar ratio are means (95%-confidence intervals)

^‡^results for IGFBP-1 and IGFBP-5 are geometric means (95%-confidence intervals).

^§^ IGFBP-1, IGFBP-5 and PAPPA estimates on a logarithmic scale were back transformed.
